# Preclinical efficacy of the novel competitive NAMPT inhibitor STF-118804 in pancreatic cancer

**DOI:** 10.18632/oncotarget.18841

**Published:** 2017-06-29

**Authors:** Jair Machado Espindola-Netto, Claudia C.S. Chini, Mariana Tarragó, Enfeng Wang, Shamit Dutta, Krishnendu Pal, Debabrata Mukhopadhyay, Mauro Sola-Penna, Eduardo N. Chini

**Affiliations:** ^1^ Laboratory of Signal Transduction and Molecular Nutrition, Department of Anesthesiology, Mayo Clinic College of Medicine, Rochester, MN, U.S.A; ^2^ Laboratório de Enzimologia e Controle do Metabolismo (LabECoM), Departamento de Biotecnologia Farmacêutica (BioTecFar), Faculdade de Farmacia, Centro de Ciencias da Saúde, Universidade Federal do Rio de Janeiro, Rio de Janeiro, Brazil; ^3^ Department of Biochemistry and Molecular Biology, College of Medicine, Mayo Clinic, Rochester, MN, U.S.A

**Keywords:** NAD, NAMPT, STF-11804, pancraetic cancer, metabolism

## Abstract

NAD salvage is one of the pathways used to generate NAD in mammals. Nicotinamide phosphoribosyltransferase (NAMPT), the rate-limiting enzyme in this pathway, uses nicotinamide (NAM) to generate nicotinamide mononucleotide (NMN). NMN is one of the main precursors of NAD synthesis in cells. Our previous study showed the importance of NAMPT in maintaining NAD levels in pancreatic ductal adenocarcinoma cells (PDAC), and that the NAMPT inhibitor FK866 decreased pancreatic cancer growth. We now tested the effect of STF-118804, a new highly specific NAMPT inhibitor, in models of pancreatic ductal adenocarcinoma. STF-118804 reduced viability and growth of different PDAC lines, as well as the formation of colonies in soft agar. In addition, STF-118804 decreased glucose uptake, lactate excretion, and ATP levels, resulting in metabolic collapse. STF-118804 treatment activated AMPK and inhibited of mTOR pathways in these cells. This effect was significantly potentiated by pharmacological AMPK activation and mTOR inhibition. Exogenous NMN blocked both the activation of the AMPK pathway and the decrease in cell viability. Panc-1 cells expressing GFP-luciferase were orthotopically implanted on mice pancreas to test the *in vivo* effectiveness of STF-118804. Both STF-118804 and FK866 reduced tumor size after 21 days of treatment. Combinations of STF-118804 with chemotherapeutic agents such as paclitaxel, gemcitabine, and etoposide showed an additive effect in decreasing cell viability and growth. In conclusion, our preclinical study shows that the NAMPT inhibitor STF-118804 reduced the growth of PDAC *in vitro* and *in vivo* and had an additive effect in combination with main current chemotherapeutic drugs.

## INTRODUCTION

NAD is crucial for cell metabolism, participating as a cofactor in redox reactions as well as being a substrate for NAD^+^-degrading enzymes such as sirtuins, poly(ADP-ribose) polymerases (PARPs), and NAD^+^ases like CD38 [1]. NAD levels are maintained via an equilibrium between its synthesis and degradation. Although NAD is synthesized via the *de novo* pathway from tryptophan, the majority of the cellular NAD synthesis in most mammalian tissues comes from the NAD salvage pathway, which uses Nicotinic Acid (NA) or nicotinamide (NAM) as main precursors [2]. Because of the high rate of NAD degradation, the conversion of NAM into nicotinamide mononucleotide (NMN) is the most effective pathway to maintain NAD levels in most tissues [3]. Nicotinamide phosphoribosyltransferase (NAMPT) is the rate-limiting enzyme responsible for this conversion, and this enzyme has been shown to be overexpressed in many types of cancer, including lung, prostate, gastric, colorectal, and pancreatic cancer [4–8].

Pancreatic ductal adenocarcinoma (PDA) is predicted to become the second leading cancer-related cause of death by 2020. This disease has an extremely poor prognosis, with current 5-year survival rate of less than 5% despite efforts to find new therapies. In most cases, surgery for tumor resection is not feasible, and chemotherapy and radiation become the only options for treatment of PDA [9, 10]. Secondary therapeutic failure and resistance to available drugs are pitfalls of chemotherapy for this type of cancer, making it necessary to find new targets and drugs that could treat PDAC [11, 12, 13].

NAMPT inhibitors have shown preclinical efficacy in a plethora of cancer models, including pancreatic cancer [8, 14, 15]. Inhibition of this rate-limiting enzyme in the synthesis of NAD causes a drop in NAD levels, ultimately leading to a reduction in cancer cell viability. Our group showed previously that NAMPT is an important metabolic target in PDAC, and FK866, a non-competitive inhibitor of this enzyme, decreases cancer cell viability *in vitro* and *in vivo* [16]. STF-118804 (STF) is a highly specific next-generation NAMPT inhibitor. In preclinical studies, STF-118804 showed an improvement in survival in an orthotopic model of high-risk acute lymphoblastic leukemia [17]. Our present work aims to evaluate the efficacy of this new inhibitor in pancreatic cancer and compare its effectiveness to FK866, a first generation drug.

To study the role of STF-118804 in pancreatic cancer, we tested the effect of this compound on cell viability and growth, as well as the metabolic effects and pathways involved in its action in cellular models of pancreatic cancer. The *in vivo* activity of this compound, in comparison with FK866, was examined using a pancreatic cancer orthotopic model. We also investigated the potential use of STF-118804 in combination with other drugs currently used in chemotherapy for pancreatic cancer.

## RESULTS

### STF-118804 decreases pancreatic cell survival and growth

**Figure 1:**
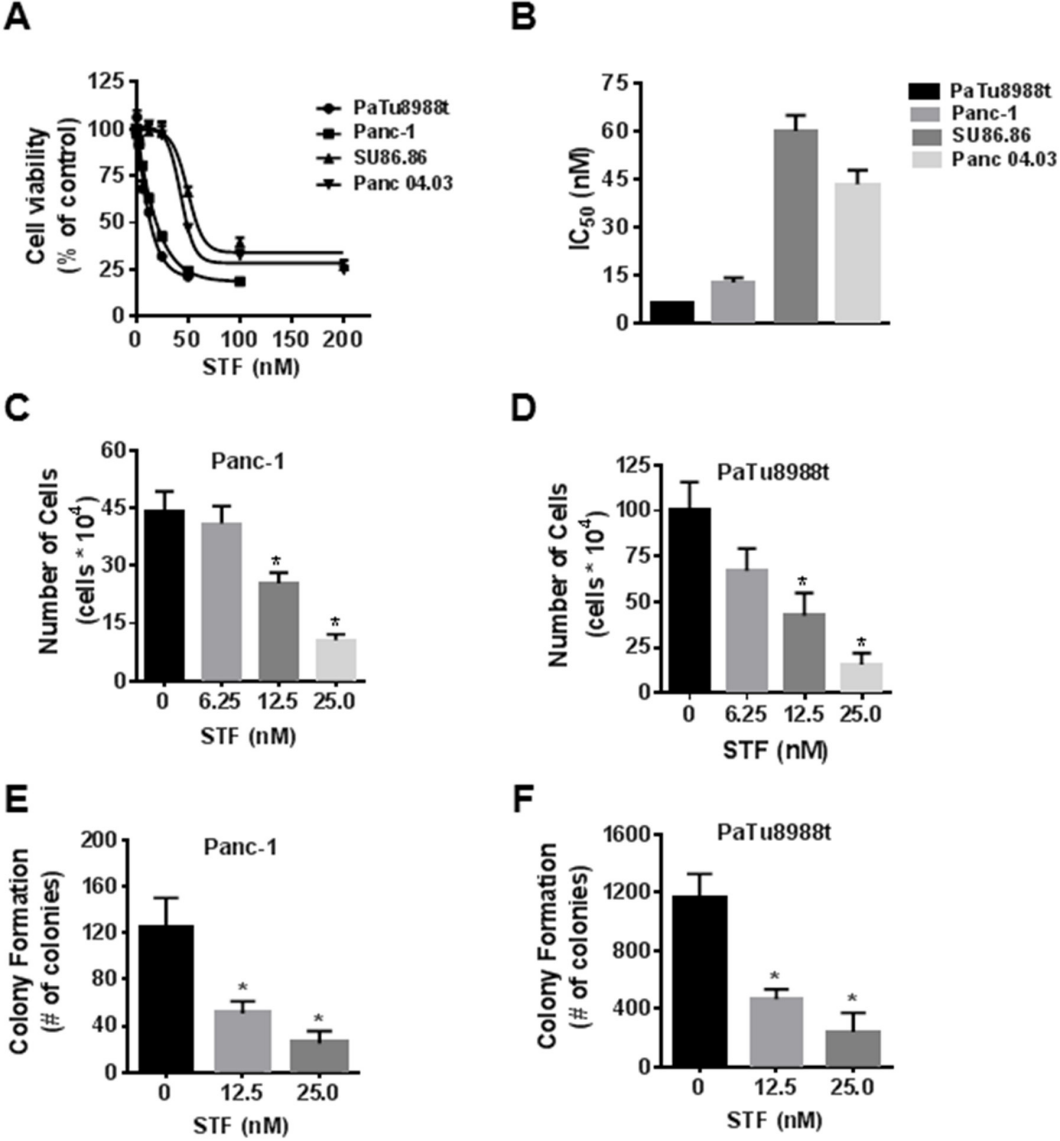
STF-118804 decreases pancreatic cancer cell survival and growth **(A** and **B)** PDAC (PaTu8988t, Panc-1, Su86.86, Panc04.03) were treated with vehicle (control) or different concentrations of the NAMPT inhibitor STF-118804 and submitted to MTT analysis 72 hours after treatment. IC_50_ is expressed in panel B. **(C** and **D)** Panc-1 and PaTu8988t cells were treated with vehicle (control) or STF-118804 for 72h and cells were counted by Trypan blue dye exclusion assay. **(E** and **F)** Panc-1 and PaTu8988t cells were grown in soft agar containing vehicle or different concentrations of STF-118804 for 14 days. Graph shows quantitative analysis of colony number. Values are mean ± SEM of at least three independent experiments performed at least in triplicate. * indicates p< 0.05.

As a first step, we assessed the effect of STF-118804 (STF) on cell viability in four PDAC lines: Panc-1, PaTu8988t, SU86.86, and Panc04.03, using the MTT assay. As we observed before for FK866, nanomolar doses of STF markedly decreased cell viability in all PDAC lines tested (Figure 1A). Panc-1 and PaTu8988t cells were more sensitive to STF, as shown by the IC_50_ in Figure 1B. Although NAD turnover is slower in non-tumor cells, NAD metabolism is important to these cells. As a control, we tested the effect of STF on viability of both human peripheral blood mononuclear cells (PBMC) and murine embryonic fibroblasts (MEFs). Both cell types were less sensitive to STF than Panc-1 and PaTu8988t (Supplementary Figure 1A and 1B). Since the MTT assay is a metabolic-based assay, we also used the trypan blue exclusion assay and sulforhodamine B assay to confirm the effect of STF on cell growth. STF decreased the number of viable cells in both assays, in nanomolar range (Figure 1C and 1D and Supplementary Figures 2A and 2B). Anchorage-independent growth is a hallmark of cancer cells and one of the best *in vitro* predictors of malignant behavior [18, 19]. STF reduced the number of colonies of both Panc-1 and PaTu8988t in soft agar in a concentration-dependent manner (Figure 1E and 1F). Our results demonstrate that STF reduces the viability of PDAC *in vitro*.

### STF-118804 induces metabolic collapse in PDAC

**Figure 2:**
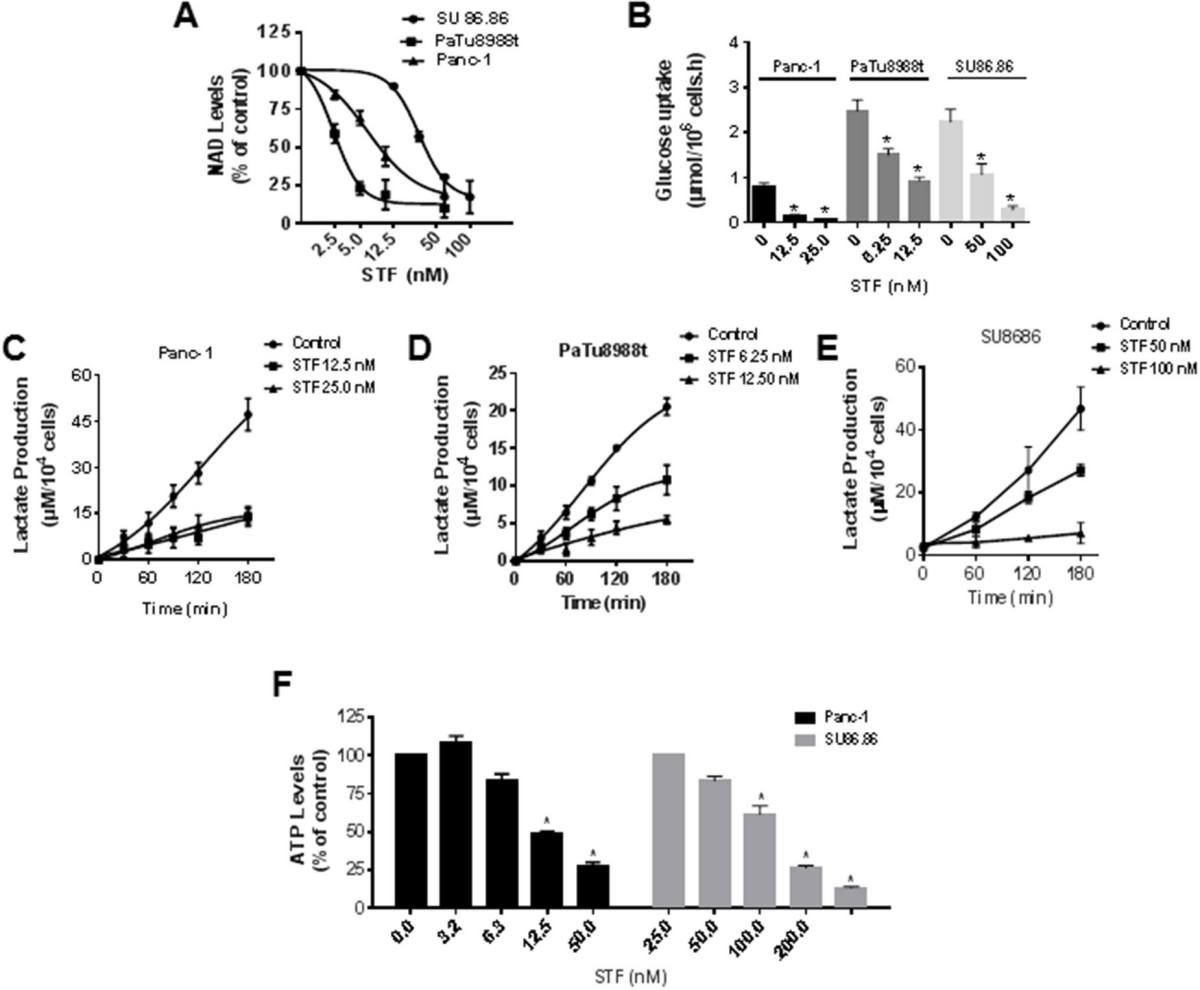
STF-118804 induces metabolic collapse in pancreatic cancer cells **(A)** NAD levels were measured in PDAC lines (Panc-1, PaTu8988t, SU86.86) treated with vehicle or different concentrations of STF-118804 for 24 hours. **(B)** PDAC lines (Panc-1, PaTu8988t and SU86.86) were treated with vehicle or different concentrations of STF-118804 for 48 hours. Changes in glucose in the media were measured after 3 hours. (**C-E)** PDAC lines (Panc-1, PaTu8988t and SU86.86) were treated with vehicle or different concentrations of STF-118804 for 48 hours and lactate release in the media was measured. **(F)** ATP levels were measured in Panc-1 and SU86.86 cells 48 hours after treatment with vehicle or different concentrations of STF-118804. Values are mean ± SEM of at least three independent experiments performed in triplicates. * indicates p< 0.05, compared to control group.

NAD is an important coenzyme regulating many reactions in metabolism and changes in NAD levels have an impact in a plethora of metabolic pathways. In order to characterize the metabolic pathways regulated by STF we first measured its effect on NAD levels in different cell lines. STF treatment reduced the levels of NAD in a concentration-dependent manner in all three PDAC lines tested (Figure 2A). Similar to what was observed on cell viability, higher concentrations of STF were needed to decrease NAD levels in SU86.86 than in Panc-1 and PaTu8988t cells.

Many cancer cells rely on high glycolytic flux, even when regular oxygen concentration is available, the so-called Warburg effect [20]. As NAD is important to the glycolytic pathway, we determined the effect of STF on glucose consumption and lactate production in sensitive cells and also in the less sensitive SU86.86 cell line. STF reduced the uptake of glucose (Figure 2B) and the release of lactate (Figure 2C-2E) in the cell media for all cell lines tested. STF caused a concentration-dependent decrease in ATP levels of Panc-1 and also more resistant cell line SU86.86 (Figure 2F). These results altogether show that STF treatment decreases glucose energy metabolism and ATP levels, and leads to metabolic collapse in PDAC.

### STF-118804 induces metabolic collapse in a time-dependent manner, promoting activation of the AMPK and inhibition of the mTOR pathway

**Figure 3:**
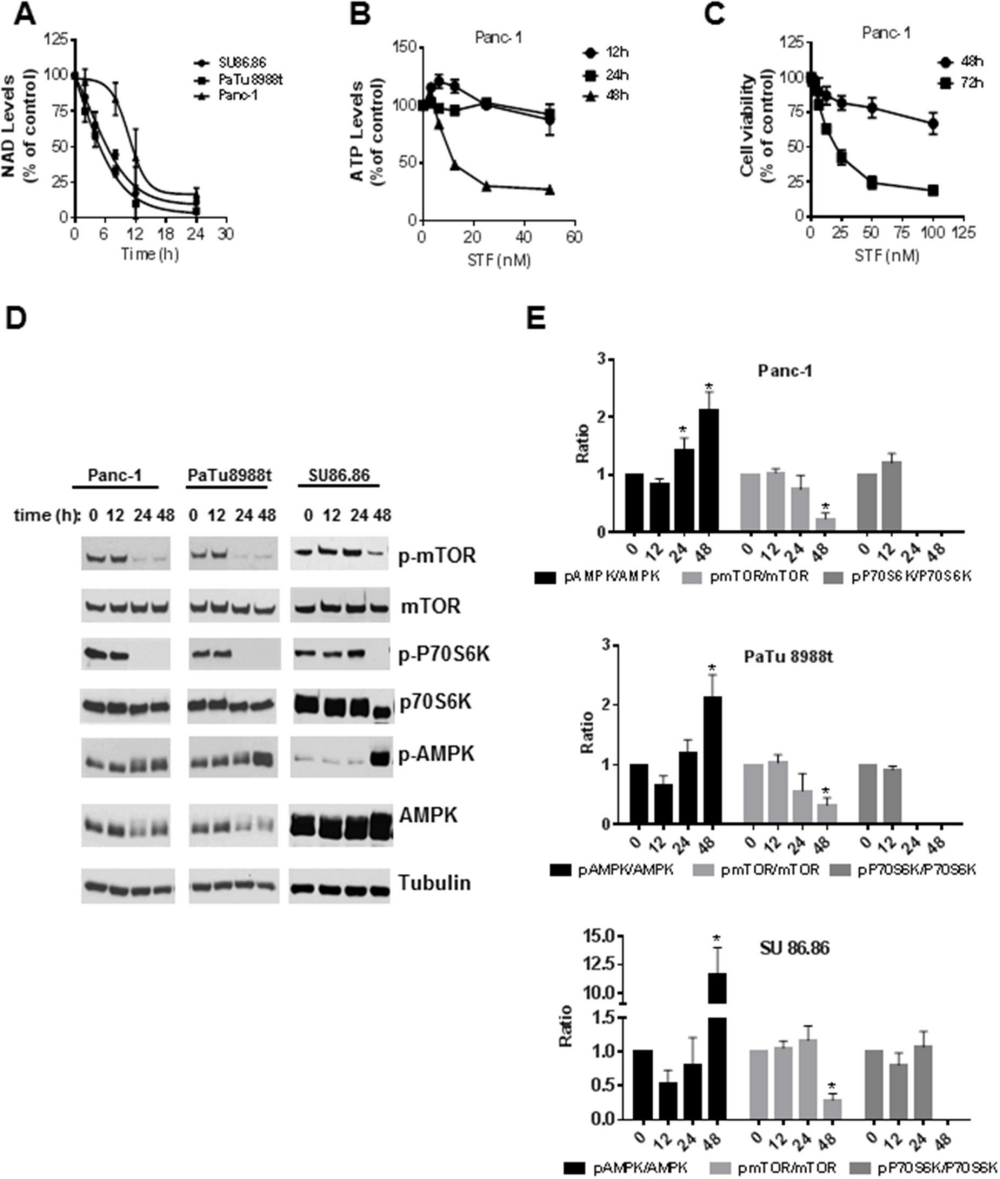
STF-118804 induction of metabolic collapse is time-dependent and promotes activation of the AMPK and inhibition of the mTOR pathway **(A)** NAD levels were measured after different time periods of treatment of PDAC lines with 25 nM (PaTu8988t and Panc-1) and 100 nM (SU86.86) STF-118804. **(B)** ATP levels were measured in Panc-1 cells treated with vehicle or different concentrations of STF-118804 at different time points. **(C)** Cell viability of Panc-1 cells was assayed 48 and 72 hours after treatment with vehicle or different concentrations of STF-118804. **(D-E)** Panc-1 and PaTu8988t cells were treated with 25 nM of STF-118804 and SU86.86 was treated with 300 nM and expression of phosphorylated proteins after different time periods was examined by immunoblotting. Values are mean ± SEM of at least three independent experiments performed at least in triplicate. * indicates p< 0.05 compared to control group.

The decrease in NAD levels observed upon treatment of PDAC with STF occurred in a time-dependent manner in all cell lines tested (Figure 3A). For PaTu8988t and SU86.86 cells, 12 hours of incubation with STF caused a reduction of around 75% in NAD levels compared to the control-treated cells. Although the curve for Panc-1 cells has a different shape, depletion of NAD was observed in all cell lines after 24 hours of treatment with STF. A time-dependent decrease in ATP levels was also observed (Figure 3B), though a significant decrease was only observed after 48 hours of treatment with the drug. Cell viability was only minimally reduced after 48 hours of incubation with STF, even at high concentrations like 100 nM (Figure 3C). On the other hand, after 72 hours of incubation with STF, even the low concentration of 12.5 nM reduced cell viability to 60% (Figure 3C).

Activation of the AMPK pathway is a marker of cell metabolic dysfunction. To test whether the STF-induced NAD depletion and metabolic collapse activates AMPK and inhibits the mTOR pathway in PDAC, we measured the phosphorylation state of members of the AMPK and mTOR signaling pathway. STF treatment for 48 hours increased the phosphorylation of AMPKα at Thr172 (Figure 3D-3E). Phosphorylation of mTOR (Ser2448) and its downstream target 70S ribosomal protein S6 kinase (P70S6K) were down regulated in all cell lines. Particularly, the phosphorylation of P70S6K was completely abolished in all cells, indicating that STF likely interferes with protein synthesis and cell growth in PDAC.

### The role of AMPK and mTOR pathway on the effect of STF-118804

**Figure 4:**
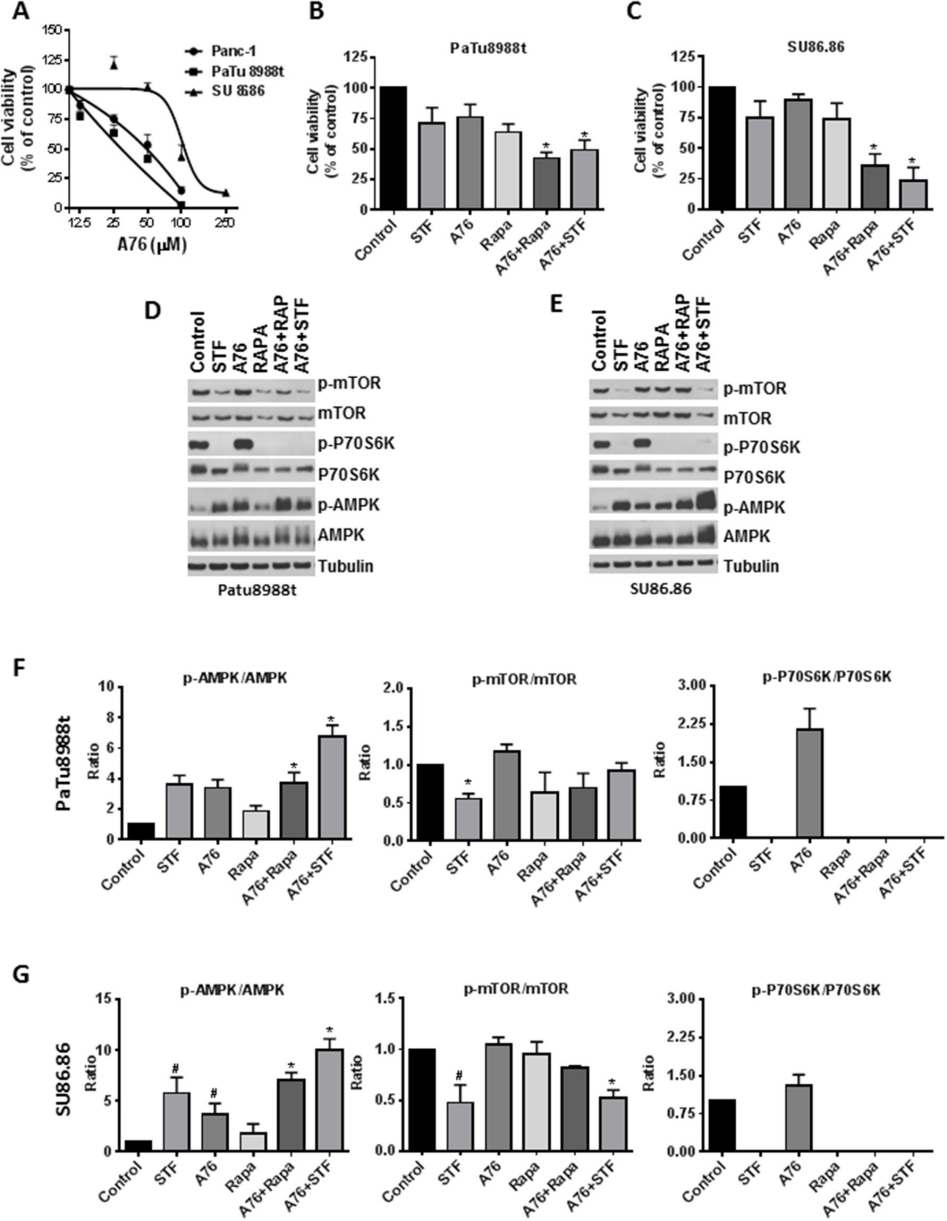
Contribution of AMPK and mTOR pathway to STF-118804 effect **(A)** Cells were treated with different concentration of the AMPK activator A769662 and its effect on cell viability was accessed after 72 h by MTT assay. **(B** and **C)** Cells were treated with either A769662 (50 μM for SU86.86 and 25 μM PaTu8688t), STF-118804 (6.125 nM for PaTu8688t and 50 nM for SU86.86) and rapamycin (200 nM for PaTu8688t and 500 nM for Su86.86) alone or in combination and assayed 72h after for cell viability by MTT assay. **(D** and **E)** Cells were treated with either A769662 (50 μM), STF-118804 (25 nM for PaTu8988 and 100 nM for Su86.86) and rapamycin (200 nM for PaTu8988t and 500 nM for SU86.86) alone or in combination and assayed for 48h and protein phosphorylation was detected by immunoblotting. **(F** and **G)** quantification of immunoblotting. Values are mean ± SEM of at least three independent experiments performed at least in triplicates. * indicates p < 0.05 for combination compared to each drug alone. # indicates p < 0.05 compared to control group.

To evaluate the role of AMPK on the effect of STF, we treated both sensitive and less sensitive cell lines with A769662 (shown A76 from here on), a highly specific AMPK activator. A76 reduced the cell viability of all cell lines tested, and SU86.86 was more resistant to its effect on cell viability (Figure 4A). As STF leads to both an activation of AMPK by phosphorylation and a decrease in phosphorylation of P70S6K, a downstream effector of mTOR, we tested the effect of A76 on phosphorylation of these proteins as well as its combination with STF and Rapamycin (Rapa) (Figure 4B-4G). Treatment with A76 leads to an increased phosphorylation of AMPK in both sensitive and non-sensitive PDAC. However, unlike STF, A76 did not decrease the phosphorylation state of P70S6K. For this reason, we tested the combination of A76 with both STF and Rapamycin and evaluated the effect on both cell viability and the phosphorylation ratio of proteins. Combination of both STF and Rapamycin with A76 lead to a decrease in cell viability that was more pronounced than each compound alone. This decrease on viability was more evident in the less sensitive cell line SU86.86 and was preceded by an increase in phosphorylation of AMPK and a complete dephosphorylation of P70S6K in both cell lines. These results suggest that both phosphorylation of AMPK and dephosphorylation of P70S6K may contribute to the loss of cell viability induced by STF.

### NMN and nicotinic acid (NA) block the effect of STF-118804 on cell viability and on the AMPK and mTOR pathways

**Figure 5:**
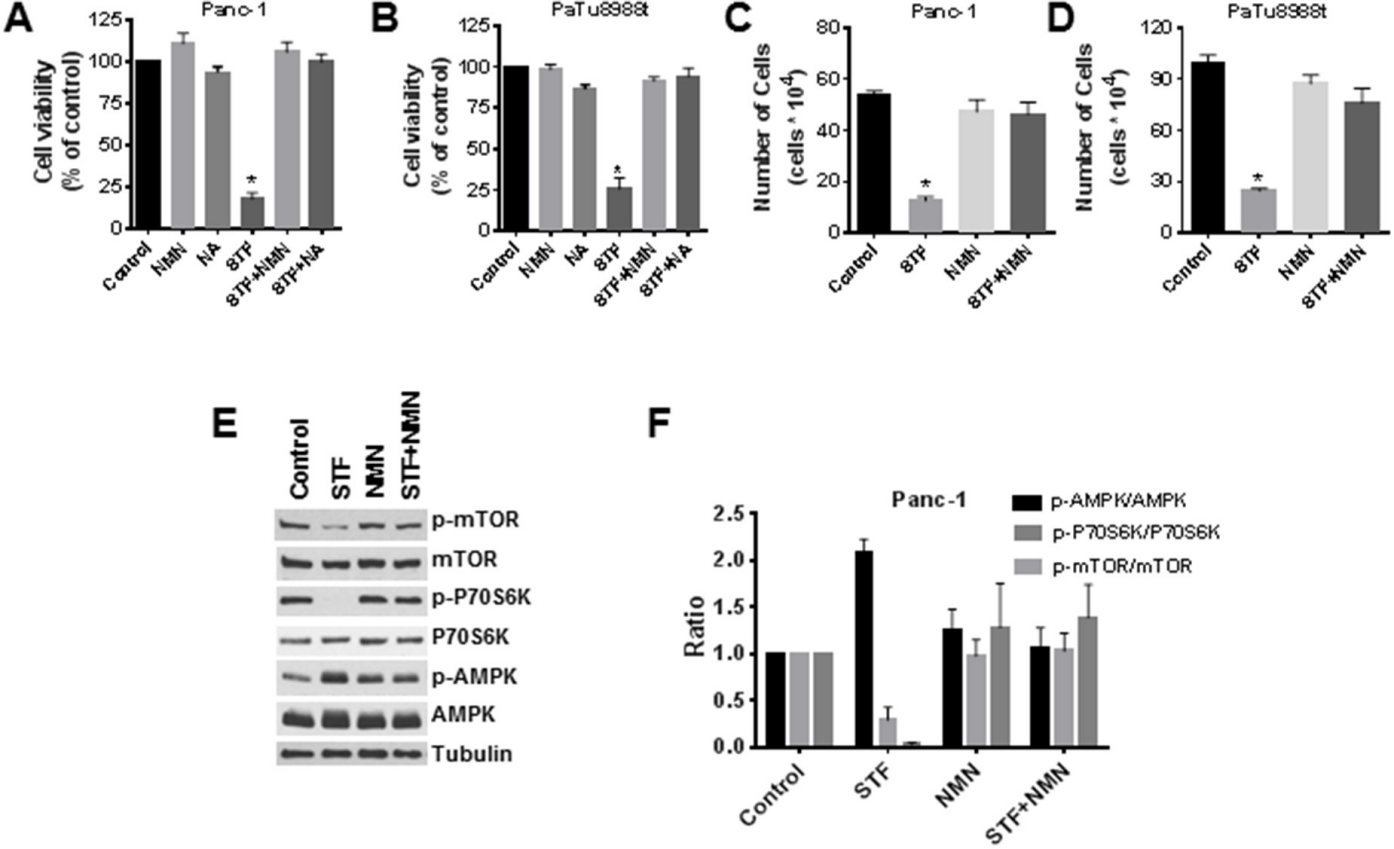
NMN and NA block the effect of STF-118804 on cell viability and on the activation of the AMPK pathway **(A** and **B)** Cells were treated with vehicle, 25 μM NMN or 25 μM NA for 6 hours before addition of 25 nM STF-118804, and viability was measured 72 hours after treatment by MTT assay. **(C** and **D)** Cells were treated with vehicle or 25 μM NMN for 6 hours before addition of 25 nM of STF-118804, and viability was determined 72 hours after treatment by Trypan blue exclusion assay. **(E** and **F)** Panc-1 cells were treated with vehicle or 25 μM NMN for 6 hours before addition of 25 nM STF-118804 for 48 hours. Expression of phosphorylated proteins was analysed by immunoblotting and quantified. Values are mean ± SEM of at least three independent experiments performed in triplicates. * indicates p< 0.05 compared to control group.

As discussed before, NAMPT is the rate-limiting enzyme responsible for the conversion of nicotinamide into NMN, which will then be converted into NAD by NMN adenylyl transferase (NMNAT) proteins in the salvage pathway. It is widely known that treatment of cells with NMN leads to an increase in NAD levels. We observed that addition of NMN blocked the effect of STF on cell viability and growth for both PaTu8988t and Panc-1 cells, using both the MTT and trypan blue exclusion assays (Figure 5A-5D). We also tested the effect of NA, a precursor of NAD in the NAD salvage pathway via the enzyme nicotinate phosphoribosyltransferase domain containing 1. Addition of NA to cells also blocked the effect of STF on cell viability (Figure 5A and 5B). To confirm that NMN regulates the effect of STF on phosphorylation status of targeted proteins, we treated cells with STF in the presence and absence of pre-incubation with NMN and measured its effects on the AMPK and mTOR pathways. Consistent with the results on the cell viability experiments, the effect of STF on the AMPK and mTOR pathway was also reversed by NMN (Figure 5E and 5F). This clearly demonstrates that the effects of STF are mediated by its effects on NAD metabolism and may be linked to metabolic collapse caused by this drug.

### STF-118804 and FK866 suppress growth of an orthotopic mouse model

**Figure 6:**
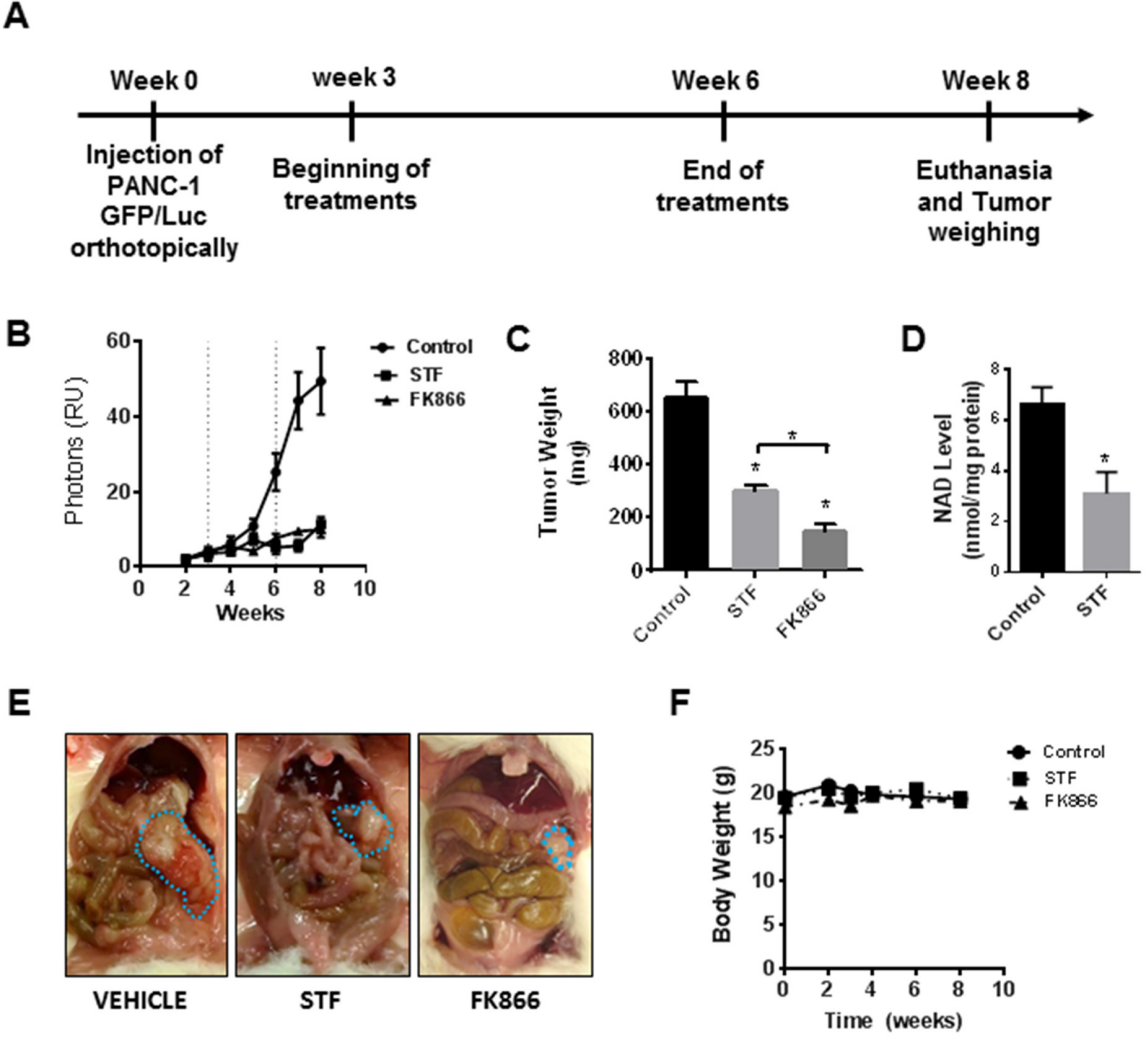
STF-118804 and FK866 are effective in suppress pancreatic tumor growth *in vivo* in an orthotopic model **(A)** Schematic representation of the treatment. **(B)** Tumor growth *in vivo* was assessed by luminescence measurements. Mice were orthotopically injected with Panc-1-GFP/Luciferase cells and randomized into three groups: vehicle (n=10), STF-118804 25 mg/kg (n=10), and FK866 15 mg/kg (n=10). Mice were treated for 3 consecutive weeks and tumor growth was followed for two more weeks without any treatment. **(C)** Tumor weight after treatments. **(D)** NAD Levels in tumors from mice treated with both vehicle and STF 25 mg/kg **(E)** Picture of orthotopic tumors at the end of the experiment. **(F)** Body weight of mice measured during the experiment. Values are mean ± SEM. * indicates p< 0.05 compared to control group.

The main aims of this study were to evaluate the STF efficacy as a treatment for pancreatic cancer, as well as to compare its efficacy with the first generation NAMPT inhibitor, FK866. Our previous study in PDAC showed that FK866 induced metabolic collapse and decreased cell viability and growth of tumor xenografts *in vivo*. Here, in order to determine the potential preclinical use of STF-118804 in pancreatic cancer, we tested both STF and FK866 in an orthotopic mouse model of PDAC. Mice were injected with Panc-1 cells containing GFP-luciferase, and after 3 weeks the drug treatments were initiated. Mice were treated for three weeks with either vehicle, STF, or FK866. After this period, drug treatment was stopped for evaluation of the remission phase and mice were sacrificed two weeks later (Figure 6A). Both drugs showed *in vivo* efficacy in this model, which was demonstrated by the reduction in luminescence (Figure 6B) and the tumor weight and size at the end of the treatment (Figure 6C and 6E). No significant toxicity was observed for these compounds either during treatment or in the remission phase. The body weight of the animals remained constant during the course of the experiment (Figure 6F). Our previous work shows that FK866 decreases NAD levels in tumors grown *in vivo*. To confirm the inhibition of Nampt *in vivo* and also the metabolic effect of STF in this mouse model, we tested NAD levels in tumors at the end of the experiment. STF treatment decreased the NAD levels in tumors derived from this orthotopic model of pancreatic cancer (Figure 6D). These results strongly support the hypothesis that STF is effective *in vivo*, even though it is less potent than FK866.

### STF-118804 sensitizes PDAC to chemotherapeutic agents

**Figure 7:**
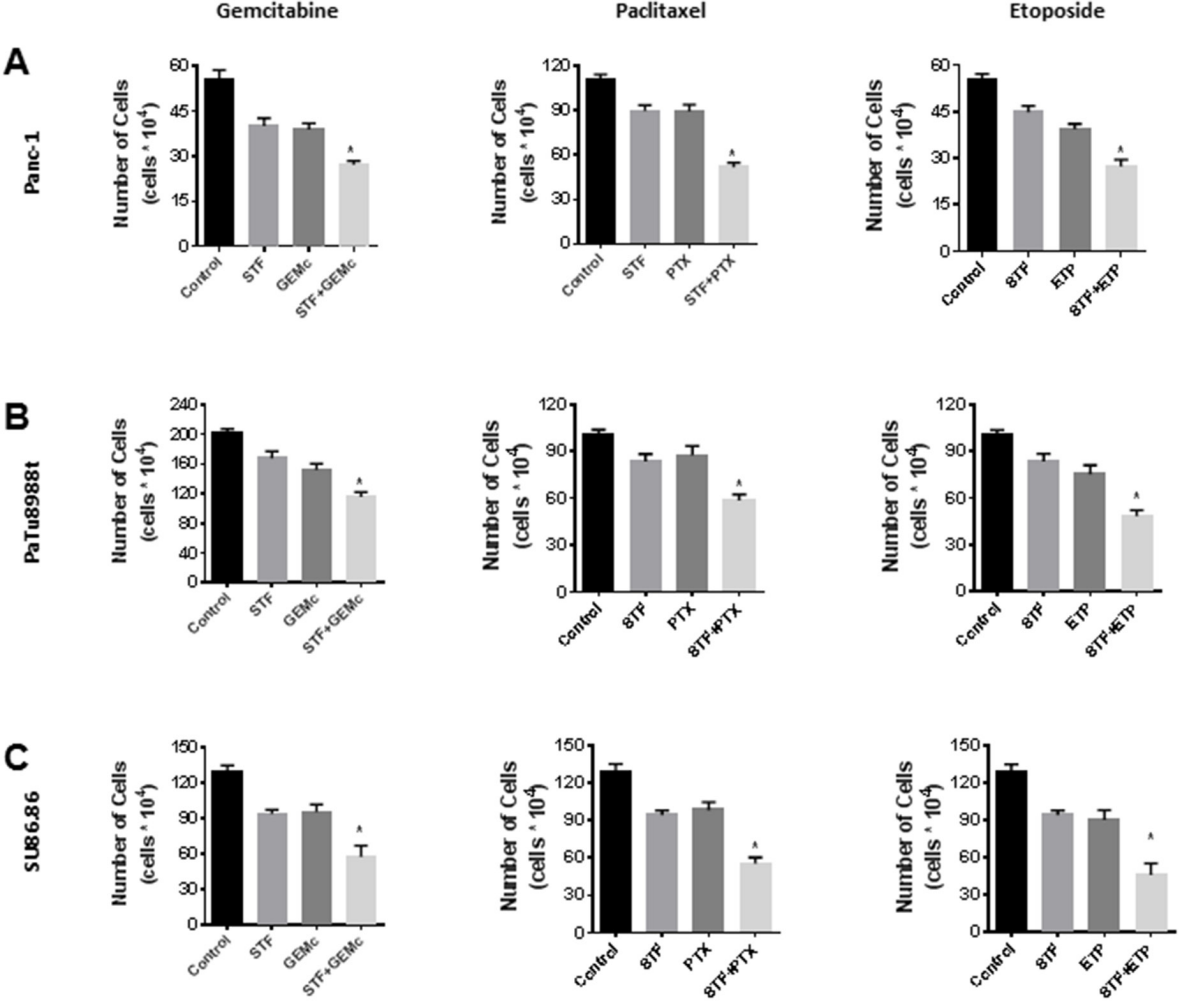
STF-118804 sensitizes PDAC to other chemotherapy treatments **(A** and **B)** Panc-1 **(A)** and PaTu8988t **(B)** and SU86.86 **(C)** cells were treated with vehicle, 12.5 nM STF-118804, and other chemotherapy drugs alone or in combination with STF-118804. Viability was measured 48h after treatments by Trypan blue dye exclusion assay. Gemcitabine was pre-incubated for 6-8 hours before addition of STF-118804, using a nucleotide-free serum. Drug concentrations: Gemcitabine 5 nM Paclitaxel 2.5 nM, Etoposide 300 nM STF-118804 6.25 nM for PaTu8988t and Panc-1 and 50 nM for SU86.86. Values are mean ± SEM of at least three independent experiments performed at least in triplicate. * indicates p< 0.05 compared to both drugs alone.

We evaluated the effect of STF in combination with other therapeutic agents used in PDAC therapy such as gemcitabine and paclitaxel. For this, we used the cell lines Panc-1 and PaTu8988t, and also the less sensitive SU86.86 cell line. Cells were treated with STF alone or in combination with other drugs, and cell viability was assessed by the trypan blue exclusion assay. The combination of STF and gemcitabine (Figure 7A-7C) shows a significant additive effect. This is a therapeutic gain, since gemcitabine has a crucial role in PDA treatment. The combination of STF with paclitaxel or etoposide also showed an increase in PDAC sensitivity to these agents. These results suggest that STF has the potential to be used as an additive therapeutic agent in patients.

## DISCUSSION

PDA is an aggressive malignancy characterized by local invasion and metastasis in the lymph nodes and distant sites. Despite recent advances, and the fact that there are some available therapies, the prognosis continues to be poor. For this reason, it is necessary to discover new more effective therapies [21]. An increasing amount of data have shown that decreasing NAD^+^ levels in many types of cancer cell lines leads to inhibition of cancer cell survival and growth [22, 23, 24]. Our group showed for the first time that NAD metabolism is an important pathway to target in PDAC and that NAMPT is crucial to maintain PDAC NAD metabolism. The NAMPT inhibitor FK866 reduced survival of PDAC both *in vivo* and *in vitro* by inducing metabolic collapse [16, 30]. A recent work showed that STF-118804, a new highly specific NAMPT inhibitor, had promising results in *in vivo* and *in vitro* preclinical models of high-risk acute lymphoblastic leukemia [17]. This molecule represents a new generation of NAMPT inhibitors with different chemical structure and properties that has potential to improve on the so-called first generation inhibitors including FK866, GMX1778, and GNE-617 [25]. The NAMPT inhibitors FK866 and GMX11778 did not show good results in phase I and II clinical trials [26, 27, 28]. Among other observations, the first generation drugs had a poor pharmacokinetic profile, with extremely rapid renal excretion or a poor oral bioavailability [17, 25]. Due to the central role of NAMPT in PDAC metabolism, the present work was aimed to obtain preclinical data for STF-118804 in pancreatic cancer models. A remarkable pharmacodynamics difference between FK866 and STF-118804 is that the first is a non-competitive inhibitor, while the second is reversible – even though both drugs as well as other Nampt inhibitors available seem to bind at the same site in the enzyme, a reversible inhibitor is clinically easier to manage when toxic effects take place.

In our study, we observed that STF decreased cell viability of all four PDAC lines tested, in nanomolar range, although we observed different sensitivity to this drug among the cell lines. Panc-1 and PaTu8988t cells exhibited a lower IC_50_ compared to SU86.86 and Panc04.03, in agreement with what we previously observed [16]. In our FK866 study we showed that the more sensitive cells, Panc-1 and PaTu8988t, are the cells that express lower levels of Nampt. Since they are the cells that have also higher sensitivity to STF, this data confirms that lower levels of Nampt may be associated with an increase in sensitivity to Nampt inhibitors, a finding that has been confirmed in other studies [16, 17, 29].

As NAD^+^ is an important coenzyme in redox reactions and it regulates activity of enzymes that use NAD as substrate, we started by determining NAD levels and the metabolic changes induced by STF treatment *in vitro*. It is known that tumor cells with proliferative phenotype rely on anaerobic glycolysis to maintain both energy status and glycolytic intermediates. Thus, important anabolic pathways are replenished to maintain macromolecule biosynthesis [30]. STF-118804, as well as FK866 and other NAMPT inhibitors, decreased glycolytic flux leading to a reversion of Warburg effect-like phenotype. Ju *et al.* showed that by either silencing NAMPT or inhibiting the NAMPT enzyme in PDAC lines, the NAD levels, glucose uptake, lactate excretion, and ATP levels decrease [30]. We and others demonstrated that FK866 treatment led to accumulation of glycolytic intermediates before the GAPDH step, an NAD-dependent dehydrogenase, and reduced production of serine and a-ketoglutarate. It has been postulated that this is the main mechanism by which NAMPT inhibitors reduce glycolytic flux and, consequently, ATP levels [14]. Taken together these results suggest that NAMPT is important for maintaining glucose metabolism. Another goal of our study was to assess the *in vivo* efficacy of STF-118804. We used a PDAC orthotopic model and compared the effect of STF and FK866 in inhibiting pancreatic cancer growth *in vivo*. Our data revealed that STF is also efficacious in inhibiting tumor growth in this model.

AMPK is an important energy sensor in the cell that responds to an increase in AMP/ATP ratio. STF induced a time-dependent phosphorylation of AMPK, and dephosphorylation of mTOR and its downstream effector ribosomal protein p70S6K. In many cell lines inhibition of mTOR by NAMPT inhibitors leads to induction of autophagy and caspase cleavage to induce apoptosis, mainly in hematological malignancies [17, 23, 32]. To evaluate the role of AMPK phosphorylation on the effect of STF, we used A76, an AMPK activator that increases the phosphorylation of the enzyme. Although A76 is known to be highly specific, we did not use concentration higher than 50 μM, since it was reported that doses higher than 100 μM cause cell death by inhibition of sodium potassium ATPase. Since A76 increased AMPK phosphorylation without inhibiting P70S6K phosphorylation, it seems that these two pathways might be acting independently in PDAC. Our results on both phosphorylation ratio and viability indicate that both activation of AMPK by phosphorylation and inhibition of P70S6K phosphorylation may be important for the effect of STF on cell viability. Unfortunately, when it comes to studying signalling pathways, the lack of highly specific activators and inhibitors limit the universality of the results obtained. The synergistic effect of A76 and rapamycin in less sensitivity cells opens a therapeutic door to test if both this combination and the combination of STF with other drugs that activate AMPK would be effective *in vivo* and a promising therapy for treatment of patients with PDA. A possible mechanism of cell death induced by NAMPT inhibition was described by Nagro *et al.* They show thatsome cells treated with NAMPT inhibitors are killed through a phenomenon called oncosis, a cell death mechanism secondary to ATP depletion and loss of membrane stability [33]. Further studies will be necessary to determine whether STF induces a similar cell death pathway.

Gemcitabine, paclitaxel, and etoposide remain important pharmacologic tools for the treatment of non-resectable PDAC. Gemcitabine is the reference regimen for advanced pancreatic cancer. Due to increased resistance to this agent, combination of drugs that could make cells more sensitive to gemcitabine is highly valuable. One of the resistance mechanisms for gemcitabine is that this drug activates HIF1a and increases glycolysis. Ju *et al.* proposed that inhibition of glycolysis by FK866 could explain why NAMPT inhibitors can be used with gemcitabine to improve pancreatic cancer treatment [31]. We observed that STF also sensitized PDAC to gemcitabine, suggesting that these two drugs may be used in a combination regimen. It had been previously shown that NAMPT-knockdown sensitized prostate cancer cells to etoposide and paclitaxel [4]. We obtained similar results in PDAC, since STF enhanced the loss of cell viability caused by both etoposide and paclitaxel. Our results suggest that NAMPT is important to PDAC survival under chemotherapeutic stress and that STF may be used in combination therapy for PDA.

Since metabolic-based therapies rely on the different role that given pathways have in normal and tumor cells, these approaches are often on the borderline of interfering with normal tissue metabolism. In clinical trials for NAMPT inhibitors the main dose-limiting side effects were thrombocytopenia and gastrointestinal toxicity. Although gastrointestinal-related effects may be attributable to the route of administration, thrombocytopenia seems to be a pharmacodynamics-related effect [25, 34]. Also, retinal toxicity was reported by Zabka *et al.* both in mice and in cell lines treated with FK866 [35]. Even though this has not been observed in clinical trials, it should be of concern as a toxicity endpoint for future trials.

NA and NMN blocked the effect of STF *in vitro*, and recent data showed that the FK866 effects were also blocked *in vivo* by the administration of these NAD precursors [17]. This suggests that Vitamin B_3_ could act as an antidote in cases of severe intoxication with NAMPT inhibitors. Further investigation in these directions is needed, and a model of *in vivo* thrombocytopenia that could help predict *in vivo* toxicity would be of great value. Given the importance of an effective therapy for PDA and its extremely poor prognosis, it is important to take into account the benefits and the risks of a therapy with NAMPT inhibitors in this disease.

In conclusion, our results together show that STF-118804 has a similar profile of action on PDAC lines than FK866. It induced loss of cell viability *in vitro*, and this was at least in part due to induction of metabolic collapse secondary to NAD and ATP depletion. STF-118804 also demonstrated *in vivo* efficacy comparable to FK866 and sensitized PDAC to current drugs approved to treat this disease. Given its considerable preclinical efficacy, STF-118804 is a promising agent to treat PDAC. Also, the results reinforce the key role of NAD metabolism and NAMPT as targets in pancreatic cancer.

## MATERIALS AND METHODS

### Cell lines

PaTu8988t, Panc-1, SU86.86, Panc04.03, and HPDE cells were either provided by Dr. D. Billadeau or obtained from American Type Culture Collection (ATCC). The pancreatic cancer cell lines possess K-ras and/or p53 mutations that were validated by DNA sequence analysis using published primers flanking mutated exon. PaTu8988t, Panc-1, and MEFs were grown in high-glucose Dulbecco’s Modified Eagle Medium (DMEM) supplemented with 10% FBS and penicillin/streptomycin (Invitrogen). SU86.86 and Panc04.03 cells were grown in RPMI-1640 medium supplemented with 10% FBS and penicillin/streptomycin. For all experiments, cells were kept in media containing 1% FBS for 18-24h before treatment, unless specified otherwise.

### Reagents and antibodies

Except when specified, all reagents and chemicals were purchased from Sigma Chemicals. AMPK, p-AMPK (Thr172), mTOR, p-mTOR (Ser2448), P70S6K, p-P70S6K (Thr389) antibodies were from Cell Signaling. STF-118804 was a gift from Dr(s) Michael Wei and Michael Cleary Stanford University. A769662 was purchased from Tocris and Santa Cruz Biotechnology.

### PBMC isolation

Blood from healthy human volunteers was collected according to IRB protocol # 06-002881. Peripheral Blood mononuclear cells (PBMC) from the blood donors were extracted by Ficoll density-gradient centrifugation. Freshly isolated cells were used for all experiments at 4 × 10^6^ cells/ml All experiments were carried out in RPMI-1640 containing 1% penicillin/streptomycin and 10% fetal bovine serum (FBS) at 37°C, 5% CO_2_ in a humidified atmosphere [22] Proliferation of PBMC was stimulated with concanavalin A.

### MTT assay

Cells were plated in 96-well plate (2-5 x 10^3^/well) in 1% FBS and treated with vehicle or different concentration of STF-118804 for 48-72h. Cell viability was determined by a standard MTT assay. IC_50_s were calculated using GraphPad Prism 6 software. The values presented represent the mean ± SEM from at least three independent experiments performed at least in triplicate.

### Trypan blue dye exclusion assay

PaTu8988t and Panc-1 cells were plated in 6-well plates (5x10^4^ and 1x10^5^, respectively) in 1% FBS and treated with vehicle or drugs for 48-72h. Cell viability was determined by standard Trypan blue dye exclusion assay.

### Sulforhodamine B assay

Sulforhodamine B assay was performed according to the protocol of the kit (Biovision Incorporated, K943)

### Soft agar colony formation assay

Cells were seeded at density of 10,000 per well in 6-well plate in 0.35% low melting agarose over a 0.6% bottom agarose layer in media containing 5% FBS and increasing concentration of STF-118804. Cell colonies were grown in humidified 5% CO_2_ incubator at 37° C. Colonies measuring 50 μm or more were counted after 14 days of culture using a cell colony counter (Gelcount, Oxford Optronix). Experiments were performed 5 times each in triplicates.

### NAD quantification

NAD levels were measured by an enzymatic cycling assay as described by us previously [36].

### ATP measurements

ATP content in cells was determined using the ATPlite Luminescence assay system from PerkinElmer and values were corrected by protein concentration measured by Bradford method.

### Glucose and lactate measurement

Glucose in supernatant was measured by a glucose colorimetric assay kit (Cayman Chemicals). Lactate levels in supernatant were measured by LDH activity assay and NADH formation at 340 nm as described before [37].

### Immunoblotting

Western blot analyses were performed using standard laboratory techniques as previously described. The films were quantified using the software ImageJ [16].

### Orthotopic xenograft model

PANC-1 cells were infected with a lentiviral reporter gene construct containing the bioluminescent reporter genes firefly luciferase and green fluorescent protein (GFP). Cells were sorted using fluorescence-activated cell sorting to obtain a pure population of cells expressing GFP/luciferase. The isolated cells (2 × 10^6^) were then orthotopically injected into the pancreas of nude mice (ages 4 to 6 weeks). As soon as bioluminescence was detectable, the mice were randomized into groups before the initiation of treatment. *In vivo* optical imaging for luciferase was done ∼10 min after i.p. injection of 3 mg n-Luciferin into each animal using a Xenogen-IVIS–cooled CCD optical system (Xenogen-IVIS). Animals were treated with IP injection of vehicle or drugs for 20 days twice a day (BID), as previously described [16, 17]. At the end of the 20^th^ day, treatment was stopped, but animals were still scanned once a week for 2 more weeks in order to look at the remission phase. Animals were randomized into three groups: (1) Control group receiving only vehicle, (2) group receiving injections of STF-118804 25 mg/kg BID and (3) group receiving injections of FK866 25 mg/kg BID. All the experiments were performed under the supervision and approval of the Institutional Animal Care and Use Committee at Mayo Clinic (protocol # A46414).

### Statistical analysis

Data are expressed as mean ± SEM from at least three independent experiments. Data were analyzed using unpaired t test and one-way and two-way ANOVA. Significance was set at P < 0.05.

## SUPPLEMENTARY MATERIALS FIGURES


