# Supplementary material for: Preclinical efficacy of the novel competitive NAMPT inhibitor STF-118804 in pancreatic cancer
Source: Oncotarget. 2017 Jun 29;8(49):85054–67. doi: 10.18632/oncotarget.18841 (PMC5689593; doi:10.18632/oncotarget.18841)
Supplement: Supplementary file 1 [file oncotarget-08-85054-s001.pdf]

Preclinical efficacy of the novel competitive NAMPT inhibitor STF-118804 in pancreatic cancer

SUPPLEMENTARY MATERIALS

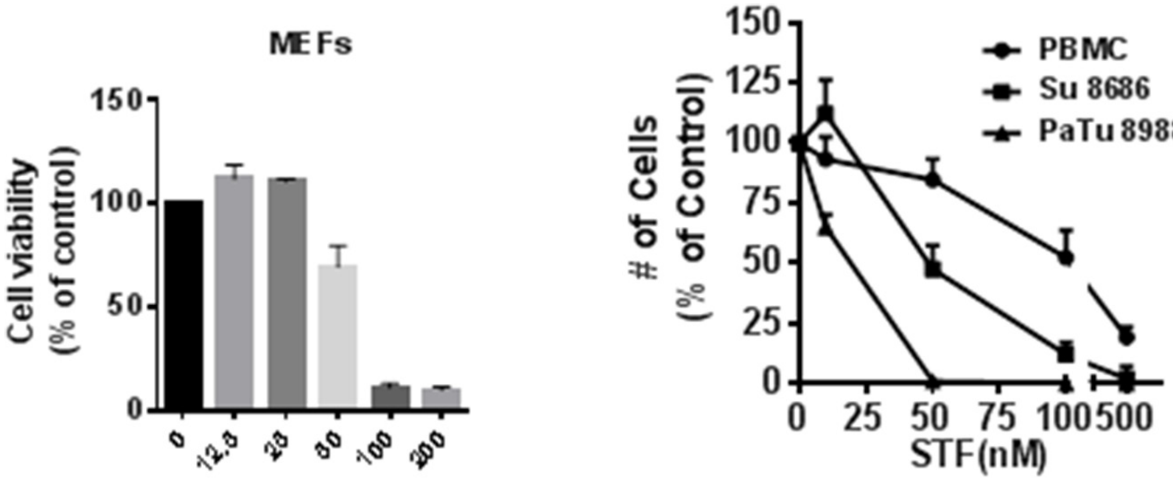

Supplementary Figure 1: STF-118804 effect on viability of mouse embryonic fibroblasts and PBMC. MEFs, PBMC and PDAC were treated with different concentrations of STF and submitted to MTT analysis 72 hours after treatment. Values are mean ± SEM of at least three independent experiments performed at least in triplicate. * indicates $p < 0.05$.

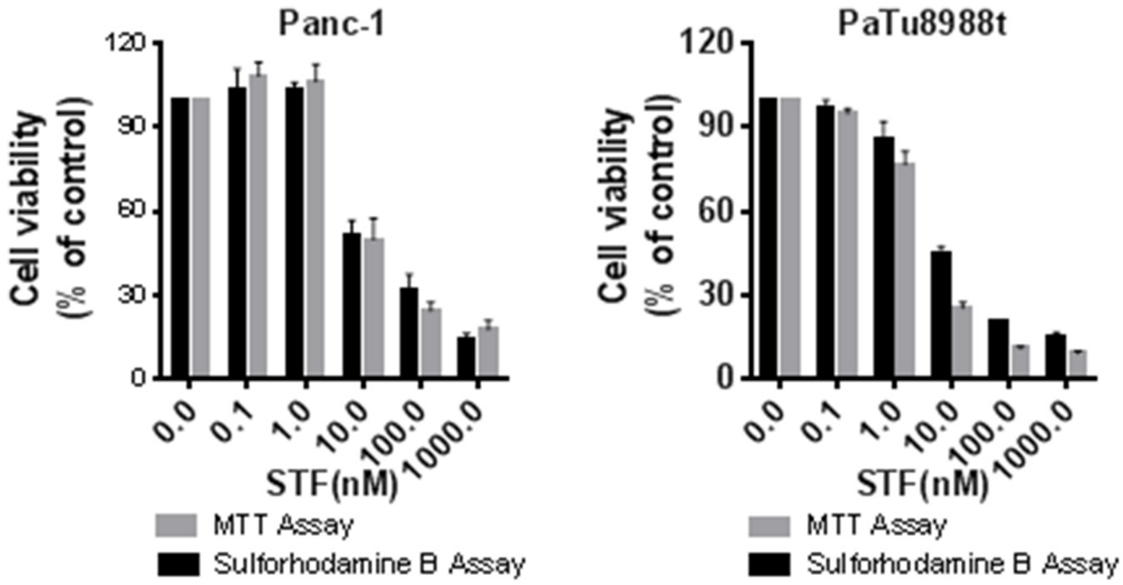

Supplementary Figure 2: Effect of STF on cell viability analyzed by two different methods. PaTu8988T and Panc-1 cells were treated with different concentration of STF side by side on the same 96 well plate and assayed by a metabolic (MTT) and a non-metabolic assay. Values are mean ± SEM of at least three independent experiments performed at least in triplicate.
